# Different Effects of Angiotensin Converting Enzyme Inhibitors on Endothelin-1 and Nitric Oxide Balance in Human Vascular Endothelial Cells: Evidence of an Oxidant-Sensitive Pathway

**DOI:** 10.1155/2008/305087

**Published:** 2008-12-01

**Authors:** Giovambattista Desideri, Davide Grassi, Giuseppe Croce, Raffaella Bocale, Sergio Tiberti, Stefano Evangelista, Stefano Necozione, Ferdinando Di Orio, Claudio Ferri

**Affiliations:** ^1^Department of Internal Medicine and Public Health, University of L'Aquila, Piazzale S. Tommasi 1, 67100 Coppito, L'Aquila, Italy; ^2^Preclinical Development Department, Menarini Ricerche S.p.A, Via Sette Santi 1, 50131 Firenze, Italy

## Abstract

Angiotensin converting enzyme inhibitors (ACE-I) are able to reduce the formation of the potent vasoconstrictor endothelin-1 and increase nitric oxide bioavailability in human vascular endothelial cells (HUVECs). We tested the effects of two sulfhydryl-containing ACE-I, zofenoprilat, and captopril, and two nonsulfhydryl containing ACE-I, enalaprilat and lisinopril, on endothelin-1/nitric oxide balance and oxidative stress in HUVECs. All the four tested ACE-I reduced endothelin-1 secretion and increased nitric oxide metabolite production by HUVECs. However, zofenoprilat (−42% after 8 hours of incubation) was more effective (*P* < .05) than enalaprilat (−25%), lisinopril (−21%), and captopril (−30%) in reducing endothelin-1 secretion. Similarly, zofenoprilat (+110% after 8 hours of incubation) was more effective (*P* < .05) than enalaprilat (+64%), lisinopril (+63%), and captopril (+65%) in increasing nitric oxide metabolite production. The effect of ACE-I on endothelin-1 and nitric oxide metabolite production is mediated by the activation of bradykinin B_2_ receptor being counteracted, at least in part, by a specific antagonist. Zofenoprilat and, to a lesser extent, captopril also reduced oxidative stress in HUVECs. In conclusion, among the four tested ACE-I, zofenoprilat was more effective in improving endothelin-1/nitric oxide balance in HUVECs likely because of its greater antioxidant properties.

## 1. INTRODUCTION

Angiotensin converting enzyme (ACE), also known as kininase II, is a bivalent dipeptidyl carboxyl metallopeptidase present both as a membrane-bound form in epithelial, neuroepithelial, and endothelial cells, including the vascular ones, and as a soluble form in different body fluid, including blood [1]. Due to its ability to cleave the C-terminal dipeptide from a number of peptides, ACE can either convert the inactive decapeptide angiotensin I to the active octapeptide angiotensin II or inactivate kinins [1]. Thus, ACE strategically modulates the balance between the vasoconstrictive and salt-retentive renin-angiotensin system and the vasodilatory and natriuretic kallikrein-kinin one [1]. As a consequence, after the initial use as antihypertensive drugs [2], ACE-inhibitors (ACE-I) rapidly became a fundamental tool also in treating congestive heart failure, left ventricular dysfunction after myocardial infarction, diabetic and nondiabetic nephropathies [2–4].

Despite of the successful use in all of the above conditions, the mechanisms responsible for the vascular benefits exerted by ACE-I are not fully understood. ACE-I are able to improve both endothelium-dependent [5] and endothelium-independent [6] vascular relaxation. However, the endothelial effects of ACE-I are not only dependent on decrease of angiotensin II formation and increase of bradykinin bioavailability [2, 5, 6]. In this regard, it has been suggested that the vascular action of ACE-I could be also related to their ability to reduce production of endothelin-1 (ET-1) [7], one of the most potent vasoconstrictor [8], through an increased nitric oxide (NO) production [7, 9] leading to a down-regulation of ET-1 gene expression [7].

In this regard, sulfhydryl containing ACE-I can act as antioxidants by scavenging superoxide anion [10] as well as nonsuperoxide radicals [11]. Since unscavenged superoxide anion quenches NO to give the pro-oxidant compound peroxynitrite [12], which is unable to down-regulate (or even up-regulates) ET-1 gene expression, sulfhydryl containing ACE-I could be particularly effective to decrease ET-1 secretion in cultured HUVECs by increasing NO production [13].

To address this topic, we compared the effects of zofenoprilat and captopril, that are two sulfhydryl containing ACE-I, with those of enalaprilat and lisinopril, two nonsulfhydryl containing ACE-I, on ET-1 secretion and NO production by human vascular endothelial cells (HUVECs). In addition, to assess the ACE-I antioxidant properties, their effects on intracellular content of the endogenous free radical scavenger reduced glutathione (GSH) [14, 15] and the generation of reactive oxygen species were also evaluated.

## 2. MATERIALS AND METHODS

### 2.1. Cells

HUVECs were harvested from fresh human umbilical cord veins cultured until the third passage as previously described [7, 16, 17]. The purity of the endothelial cell monolayer was confirmed by their cobblestone morphological pattern and by cell staining with a monoclonal antibody specific for von Willebrand factor [17]. Newly confluent cells in culture medium were lifted with trypsinization; the trypsin was inhibited with 20% foetal calf serum, and cells were washed in culture medium. After 10 minutes of centrifugation (1100 rpm, 20°C), the supernatant was removed and HUVECs were resuspended in culture medium (3 mL) and then used for the experiments.

HUVECs were incubated either with zofenoprilat (the active form of zofenopril), or enalaprilat (the active form of enalapril), or lisinopril or captopril for various times up to 24 hours. The above experiments were repeated in the presence of either bradykinin, or Des-Arg^9^-[Leu^8^]-BK, that is, a bradykinin B_1_ receptor antagonist, or D-Arg-[Hyp^3^, Thi^5,8^, D-phe^7^]-BK, that is, a bradykinin B_2_ receptor antagonist. Finally, experiments were also repeated in the presence of the NO synthase competitive inhibitor N^*ω*^-nitro-L-arginine methyl ester (L-NA).

Zofenoprilat was obtained from Menarini Ricerche SpA, Firenze, Italy. Angiotensin II was purchased by Clinalfa (Laufelfingen, Switzerland). The other reagents were purchased by Sigma (St Louis, Mo, US). If it is not otherwise specified, all the tested substances have been added to culture medium to a final concentration of 10^−8^  M, a concentration that fully inhibited the human recombinant ACE for all the antagonists under study [18].

### 2.2. Endothelin-1

The peptide was assayed as previously described [16]. In brief, the culture medium derived from each well was centrifuged at 3.000 rpm for 10 minutes. The supernatant was subsequently freeze-dried, reconstituted in starting high performance liquid chromatography buffer, injected onto C_18_ columns (Pharmacia, Uppsala, Sweden), and eluted over 70 minutes using a linear gradient of 15–75% acetonitrile/0.1% trifluoroacetic acid in water. Fractions were collected each minute and evaporated before reconstitution in assay buffer (50 mmol/L phosphate buffer, pH 7.4, containing 0.9% NaCl, 0.05% NaN_3_, and 0.5% bovine serum albumin). Endothelin-1 immunoreactivity was then assayed on reconstituted samples by a sensitive radioimmunoassay (Peninsula Laboratories, Belmont, Calif, USA). Interassay and intra-assay variations were <10%. Cross-reactivity of the ET-1 antibody with endothelin-2 and endothelin-3 was <7%, according to the supplier.

### 2.3. Nitric oxide

NO production by HUVECs was assessed by evaluating the concentration of NO metabolite (NO_*x*_), that is, nitrite plus nitrate, in culture medium. Briefly, NO_*x*_ concentrations were evaluated by colorimetric detection of nitrite after conversion of all sample nitrate to nitrite (Assay Design Inc., Ann Arbor, Mich, USA) as previously described [9].

### 2.4. Measurements of intracellular glutathione redox status and oxidative stress

Intracellular glutathione (GSH) concentration was measured according to the method previously described by our group [15]. In brief, 2 × 10^6^ HUVECs were firstly diluted in 1 mL isotonic saline + HCl (10 mmol/L) and then lysed in acetone, thawed four times, and centrifuged for 15 minutes at 4°C. Supernatants were deproteinized with 10% 5-sulfosalicylic acid and used for total GSH determination, that is, glutathione (GSH) + GSH disulphide (GSSG), by the enzymatic method described by Anderson [19]. For GSSG determination, 0.1 mL deproteinized supernatants were treated with 2 *μ*L 2-vinylpyridine, neutralized with triethanolamine at a final pH of 6.5 and assayed after 1 hour incubation. Then, endothelial cell GSH content was calculated by subtracting GSSG from total intracellular GSH concentrations.

Intracellular oxidative stress was measured at baseline and after incubation with tumor necrosis factor (TNF)*α* according to Wu and Juurlink’s method [14]. In brief, cultured HUVECs were loaded with the permeable agent 5-(6)-carboxy-2′-7′-dichlorodihydrofluorescein (DCHF) ester for 60 minutes. In the presence of intracellular esterases permeable DCHF ester is converted to its impermeable counterpart. This latter is oxidized to the fluorescent DCF by strong oxidants such as hydroxyl radicals [15, 20]. Then, intracellular oxidative stress was quantified by monitoring DCF content in HUVECs with fluorimeter with excitation at 495 nm and emission at 525 and expressed as percent of control.

### 2.5. Statistical analysis

Changes of the assessed parameters were analyzed by paired *t*-test. Multiple comparisons were analyzed by ANOVA followed by post hoc analysis with Bonferroni test. Statistical significance was considered as *P* < .05. Data are given as the mean ± SD of four experiments.

## 3. RESULTS

### 3.1. ACE-I counteracts endothelin-1 secretion in HUVECs: evidence of different pathway

**Figure 1:**
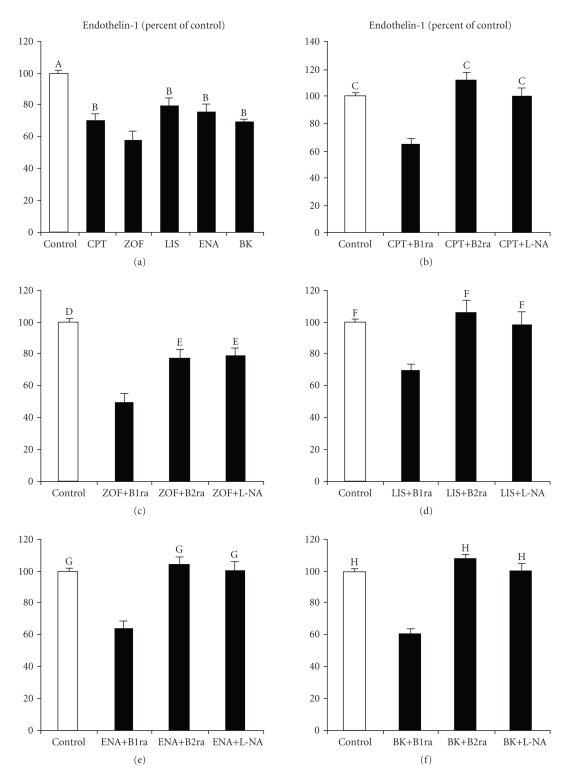
Effects of 10^−8^  M captopril (CPT, (a) and (b)), zofenoprilat (ZOF, (a) and (c)), lisinopril (LIS, (a) and (d)), enalaprilat (ENA, (a) and (e)) and bradykinin (BK, (a) and (f)) on endothelin-1 secretion (expressed as % of control) by vascular endothelial cells derived from umbilical cord vein after 8 hours of incubation both alone and in the presence of either Des-Arg^9^-[Leu^8^]-BK, that is, a bradykinin B_1_ receptor antagonist (B1ra, 10^−6^  M), or D-Arg-[Hyp^3^, Thi^5,8^, D-phe^7^]-BK, that is, a bradykinin B_2_ receptor antagonist (B2ra, 10^−6^  M), or the NO synthase competitive inhibitor N^*ω*^-nitro-L-arginine methyl ester (L-NA, 3 × 10^−3^  M). (A) *P* < .0008 or less versus CPT, ZOF, LIS, ENA, and BK; (B) *P* < .04 or less versus ZOF; (C) *P* < .0005 or less versus CPT+B1ra; (D) *P* < .0003 or less versus ZOF+B1ra, ZOF+B2ra, and ZOF+L-NA; (E) *P* < .002 versus ZOF+B1ra; (F) *P* < .0001 versus LIS+B1ra; (G) *P* < .0002 or less versus ENA+B1ra; (H) *P* < .0001 versus BK+B1ra.

All tested ACE-I at 10^−8^  M reduced spontaneous ET-1 secretion by HUVECs to a level which was similar to that induced by bradykinin (see Figure 1(a)). Preincubation with the bradykinin B_2_ receptor antagonist D-Arg-[Hyp^3^, Thi^5,8^, D-phe^7^]-BK but not the bradykinin B_1_ receptor antagonist Des-Arg^9^-[Leu^8^]-BK both at 10^−6^  M abolished the inhibitory effect of ACE-I on ET-1 secretion by HUVECs. These data suggested that bradykinin B_2_ receptor stimulation was involved in the inhibitory effect of the four tested ACE-I on ET-1 secretion. Although all the four tested drugs counteracted spontaneous ET-1 secretion by HUVECs, zofenoprilat was more effective than the other ACE-I in this setting. In addition, the inhibitory effect of zofenoprilat on ET-1 secretion was only partially counteracted by bradykinin B_2_ receptor inhibition (see Figure 1(c)). This finding indicated that bradykinin B_2_ receptor stimulation does not represent the only pathway involved in the inhibitory effect of zofenoprilat on ET-1 production by HUVECs.

### 3.2. Increased NO availability is responsible for the inhibitory effect of ACE-I on endothelin-1 secretion by HUVECs

**Figure 2:**
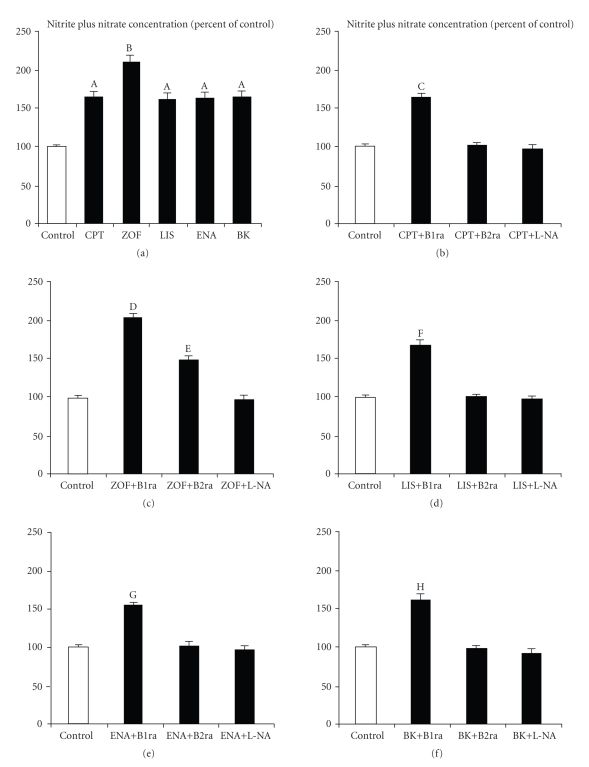
Effects of 10^−8^  M captopril (CPT, (a) and (b)), zofenoprilat (ZOF, (a) and (c)), lisinopril (LIS, (a) and (d)), enalaprilat (ENA, (a) and (e)), and bradykinin (BK, (a) and (f)) on nitric oxide production, as evaluated by nitrite plus nitrate concentrations in culture medium (expressed as % of control) by vascular endothelial cells derived from umbilical cord vein after 8 hours of incubation both alone and in the presence of either Des-Arg^9^-[Leu^8^]-BK, that is, a bradykinin B_1_ receptor antagonist (B1ra, 10^−6^  M), or D-Arg-[Hyp^3^, Thi^5,8^, D-phe^7^]-BK, that is, a bradykinin B_1_ receptor antagonist (B2ra, 10^−6^  M), or the NO synthase competitive inhibitor N^*ω*^-nitro-L-arginine methyl ester (L-NA, 3 × 10^−3^  M). (A) *P* < .0001 versus control; (B) *P* < .02 or less versus CPT, LIS, ENA, and BK and *P* < .0001 versus control; (C) *P* < .0003 or less versus control, CPT+B2ra and CPT+L-NA; (D) *P* < .0001 versus control and ZOF+L-NA and *P* < .003 versus ZOF+B2ra; (E) *P* < .003 versus control and ZOF+L-NA; (F) *P* < .0004 or less versus control, LIS+B2ra and LIS+L-NA; (G) *P* < .0006 or less versus control, ENA+B2ra and ENA+L-NA; (H) *P* < .0001 versus control, BK+B2ra and BK+L-NA.

All tested ACE-I at 10^−8^  M and bradykinin at 10^−8^  M significantly increased spontaneous NO_*x*_ concentrations in culture medium (see Figure 2(a)). ACE-I and bradykinin related changes in NO_*x*_ concentrations were counteracted by previous incubation of HUVECs with the bradykinin B_2_ receptor antagonist D-Arg-[Hyp^3^, Thi^5,8^, D-phe^7^]-BK at 10^−6^  M (see Figures 2(b)–2(f)) while the bradykinin B_1_ receptor antagonist Des-Arg^9^-[Leu^8^]-BK at 10^−6^  M was completely ineffective in this setting (see Figures 2(b)–2(f)). The preincubation of HUVECs with the NO synthase inhibitor L-NA at 3 × 10^−3^  M completely abolished the effects of ACE-I and bradykinin on NO_*x*_ production (see Figures 2(b)–2(f)). In addition, L-NA also counteracted the inhibitory effect of ACE-I on ET-1 production (see Figures 1(b)–1(f)). These data suggest that increased NO production plays a pivotal role in the inhibitory effect of ACE-I on endothelin-1 secretion by HUVECs. Although all four tested ACE-I were effective in increasing NO_*x*_ concentrations in culture medium, this effect was more evident in the presence of zofenoprilat (see Figure 2(a)). In addition, bradykinin B_2_ receptor inhibition was only partially effective in counteracting zofenoprilat-induced increment of NO_*x*_ concentrations in culture medium (see Figure 2(c)).

### 3.3. ACE-I reduces intracellular oxidative stress and increases GSH content

**Figure 3:**
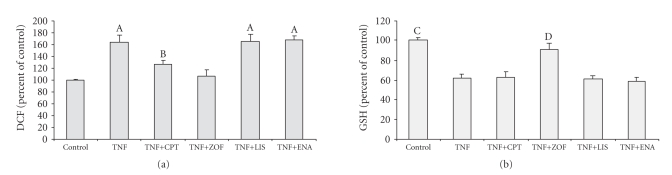
Effects of 10^−8^  M captopril (CPT), zofenoprilat (ZOF), lisinopril (LIS), and enalaprilat on TNF*α*-induced intracellular oxidative stress as evaluated by dichlorofluorescein (DCF, (a)) and glutathione (GSH, (b)) content. (A) *P* < .01 or less versus control and TNF+ZOF and *P* < .002 versus TNF+CPT; (B) *P* < .0001 versus control and *P* < .01 versus TNF+ZOF. (C) *P* < .0001 versus TNF, TNF+CPT, TNF+LIS, and TNF+ENA and *P* < .02 versus TNF+ZOF; (D) *P* < .0004 or less versus TNF, TNF+CPT, TNF+LIS, and TNF+ENA.

HUVECs preincubation with 10^−8^  M of zofenoprilat and captopril, but not with enalaprilat and lisinopril, was resulted in a significant decrease of TNF*α*-stimulated generation of reactive oxygen species (see Figure 3(a)). Although both sulfhydryl containing ACE-I reduced TNF*α*-stimulated reactive oxygen species generation in cultured HUVECs, zofenoprilat was more effective than captopril in this setting (see Figure 3(a)). In keeping with this, zofenoprilat but not captopril, lisinopril, and enalaprilat significantly protected HUVECs against the GSH decrease observed after incubation with TNF*α* (see Figure 3(b)).

## 4. DISCUSSION

The ability of ACE-I to counteract ET-1 production by endothelial cells [7, 9] has been proposed as a relevant contributor to the well-known vascular protective effects exerted by ACE-inhibition [7, 21]. Indeed, although tonic ET-1 production by endothelial cells physiologically contributes to vascular tone [21, 22], this peptide has per se all the biological potential to contribute to the onset and progression of atherosclerotic vascular damage [8, 21]. The current report provides evidence that ACE-I, tested at concentrations that fully inhibited the ACE, do not share in common similar efficacy in counteracting ET-1 release from vascular endothelial cells. Indeed, we found that zofenoprilat was more effective than captopril, lisinopril, and enalaprilat in reducing ET-1 secretion from cultured HUVECs. In addition, our data demonstrate that different intracellular pathways are involved in the inhibitory effects of the four tested ACE-I on ET-1 secretion. In this context, it has been previously demonstrated that the inhibitory effect of ACE-I on ET-1 production by HUVECs is due to a bradykinin B_2_ receptor-mediated increase in NO production by HUVECs [7, 9].

As known, oxygen derived free radical can inactivate NO [12]. In turn, NO represents a barrier against oxidants such as unscavenged superoxide anion [23]. Thus, it is reasonable to speculate that the greater effects observed with zofenoprilat in reducing ET-1 secretion and increasing NO production by cultured HUVECs might have been due to its antioxidant properties [11, 24]. In keeping with this, zofenoprilat and at lesser extent captopril, but not lisinopril and enalaprilat, were able to decrease generation of reactive oxygen species induced by TNF*α* in HUVECs. Further, zofenoprilat but not the two nonsulfhydryl containing ACE-I lisinopril and enalaprilat blunted the GSH decrease in HUVECs induced by TNF*α*. Since sulfhydryl containing ACE-I are supposed to act as antioxidants as the endogenous free radical scavenger GSH [2, 10, 11], both these findings suggest that the sulfhydryl group can be the responsible for the effect of zofenoprilat in reducing ET-1 production, that is, because of sulfhydryl-related scavenging capability and the consequent decrease in NO inactivation by endogenous oxidants. In agreement with this hypothesis, Cominacini et al. [24] demonstrated that zofenoprilat, but not enalapril, protected the intracellular proinflammatory pleiotropic mediator nuclear factor *κ*B against oxidant-induced activation and was able to spare GSH from consumption induced by oxLDL. Likewise, sulfhydrylic ACE-I have been reported to protect cultured endothelial cells against the damage induced by both superoxide and nonsuperoxide radicals [11] and to decrease LDL susceptibility to oxidation in hypertensive patients [25, 26].

However, the presence of a sulphydryl group in zofenoprilat molecule does not completely explain our findings. Indeed, the other sulfhydryl containing ACE-I tested in our study, captopril, was mildly but not significantly more effective than lisinopril and enalaprilat in our tests. These data agree with previous findings obtained in leucocytes and endothelial cells, demonstrating that captopril poorly scavenged newly generated superoxide anion [27, 28]. In this regard, zofenoprilat displays higher lipophilicity than captopril, suggesting it could exert more pronounced intracellular effects [18]. Worth mentioning in this regard, the recent evidence by Soardo et al. [29] demonstrating a stronger inhibitory effect of zofenoprilat on alcohol-induced ET-1 production by endothelial cells in comparison to carvedilol, a beta adrenoceptor blocker with known antioxidant activity [30].

In conclusion, the sulfhydryl containing ACE-I zofenoprilat, that is, the active drug of the prodrug zofenopril [18], was more effective than the nonsulfhydryl containing ACE-I lisinopril and enalapril and the sulfhydryl containing one captopril in reducing ET-1 secretion by cultured HUVECs and improving NO bioavailability. These findings likely reflect different antioxidant power between the four tested ACE-I. Since both increased ET-1 production and decreased NO bioavailability are deeply involved in the pathophysiology of atherosclerosis [22], reciprocal changes in ET-1 and NO production by the vascular endothelium could contribute to the benefits deriving from clinical use of ACE inhibitors [2]. The presence of a sulfhydryl group confers to ACE-I some ancillary properties, such as greater protection against LDL oxidation [26] and nuclear factor *κ*B activation [24], scavenging of superoxide anion [10] and nonsuperoxide radical [11], and, as demonstrated in this study, more pronounced favourable effects on ET-1/NO balance in vascular endothelial cells. Whether or not these endothelial effects of zofenoprilat could contribute to the observed cardiovascular benefits deriving from zofenopril treatment [31] remains to be elucidated.
